# Supplementary material for: Molecular Characterisation of a Novel and Highly Divergent Passerine Adenovirus 1
Source: Viruses. 2020 Sep 17;12(9):1036. doi: 10.3390/v12091036 (PMC7551158; doi:10.3390/v12091036)
Supplement: Supplementary file 1 [file viruses-12-01036-s001.pdf]

viruses

MDPI

Article

Molecular Characterisation of a Novel and Highly Divergent Passerine Adenovirus 1

Ajani Athukorala[1], Jade K. Forwood[2], David N. Phalen[3,4] and Subir Sarker[1]*

[1] Department of Physiology, Anatomy and Microbiology, School of Life Sciences, La Trobe University, Melbourne, VIC 3086, Australia; a.athukorala@latrobe.edu.au

[2] School of Biomedical Sciences, Charles Sturt University, Wagga Wagga, NSW 2678, Australia; jforwood@csu.edu.au

[3] Sydney School of Veterinary Science, University of Sydney, Camden, NSW 2570, Australia; david.phalen@sydney.edu.au

[4] Schubot Exotic Bird Health, Texas A&M College of Veterinary Medicine and Biomedical Sciences, College Station, TX 77843-4467, USA.

*Correspondence: s.sarker@latrobe.edu.au; Tel: +61 3 9479 2317; Fax: +61 3 9479 1222.

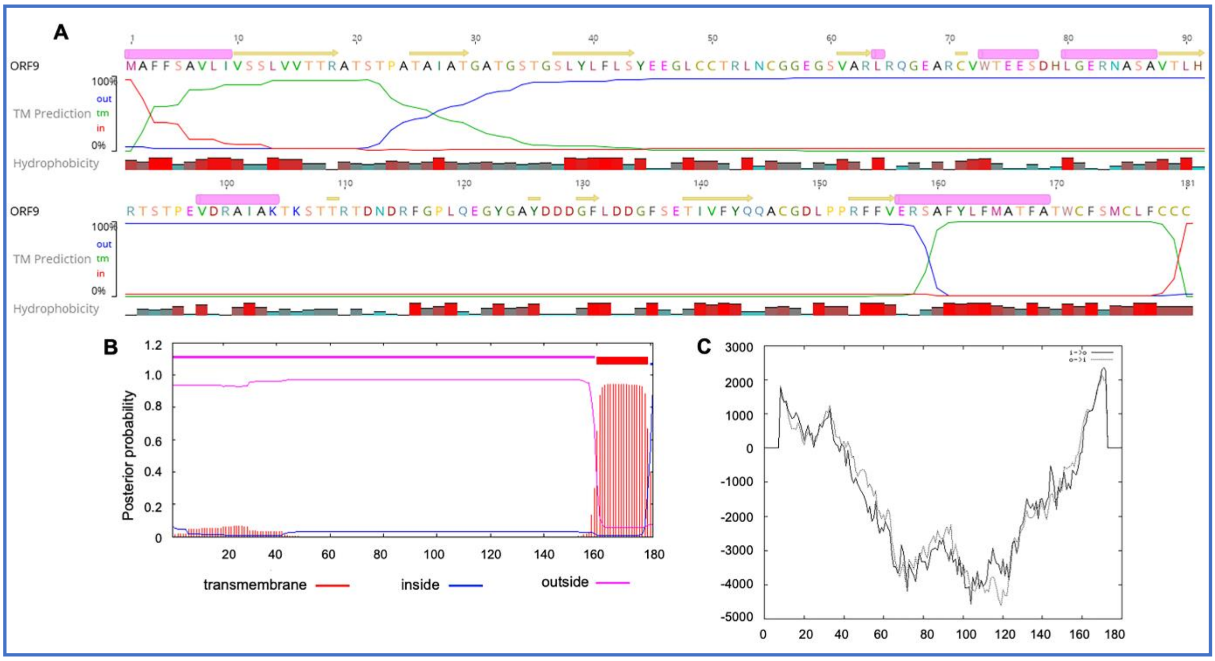

Figure S1. Predicted structure of the unique PaAdV1-ORF09. (**A**) prediction of transmembrane helices (TMHs) in unique PaAdV1-ORF10 gene using EMBOSS 6.5.7 tool in Geneious (version 10.2.2) (**A**), TMHMM (**B**) and TMpred (**C**). All the programs consistently predicted two TMHs. (**A**) TMHs detected by EMBOSS also showed the presence of alpha-helices within TMHs predicted region that has been dominated by highly hydrophobic residue (red colour). (**B** and **C**) The X-axis represents the position of residue, whereas Y-axis represents the posterior probability (**B**) and scores (above 500 are considered significant) (**C**) for the predicted TMHs. (**C**) solid and dashed black lines indicated protein orientation as inside to outside, and outside to inside, respectively.

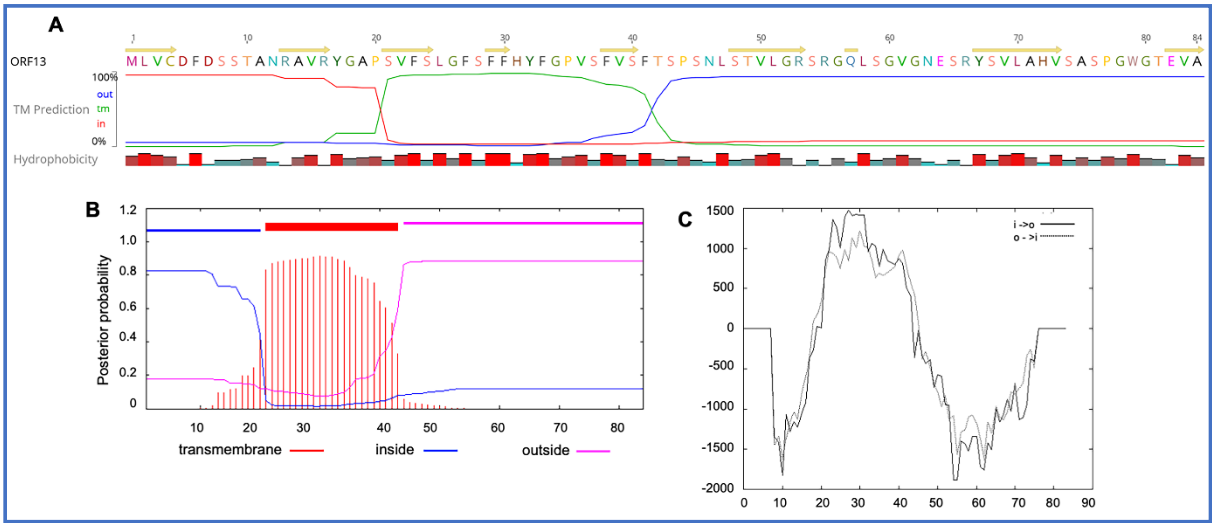

Figure S2. Predicted structure of the unique PaAdV1-ORF13. (**A**) prediction of transmembrane helices (TMHs) in unique PaAdV1-ORF10 gene using EMBOSS 6.5.7 tool in Geneious (version 10.2.2) (**A**), TMHMM (**B**) and TMpred (**C**). All the programs consistently predicted two TMHs. (**A**) TMHs detected by EMBOSS also showed the presence of alpha-helices within TMHs predicted region that has been dominated by highly hydrophobic residue (red colour). (**B** and **C**) The X-axis represents the position of residue, whereas Y-axis represents the posterior probability (**B**) and scores (above 500 are considered significant) (**C**) for the predicted TMHs. (**C**) solid and dashed black lines indicated protein orientation as inside to outside, and outside to inside, respectively.

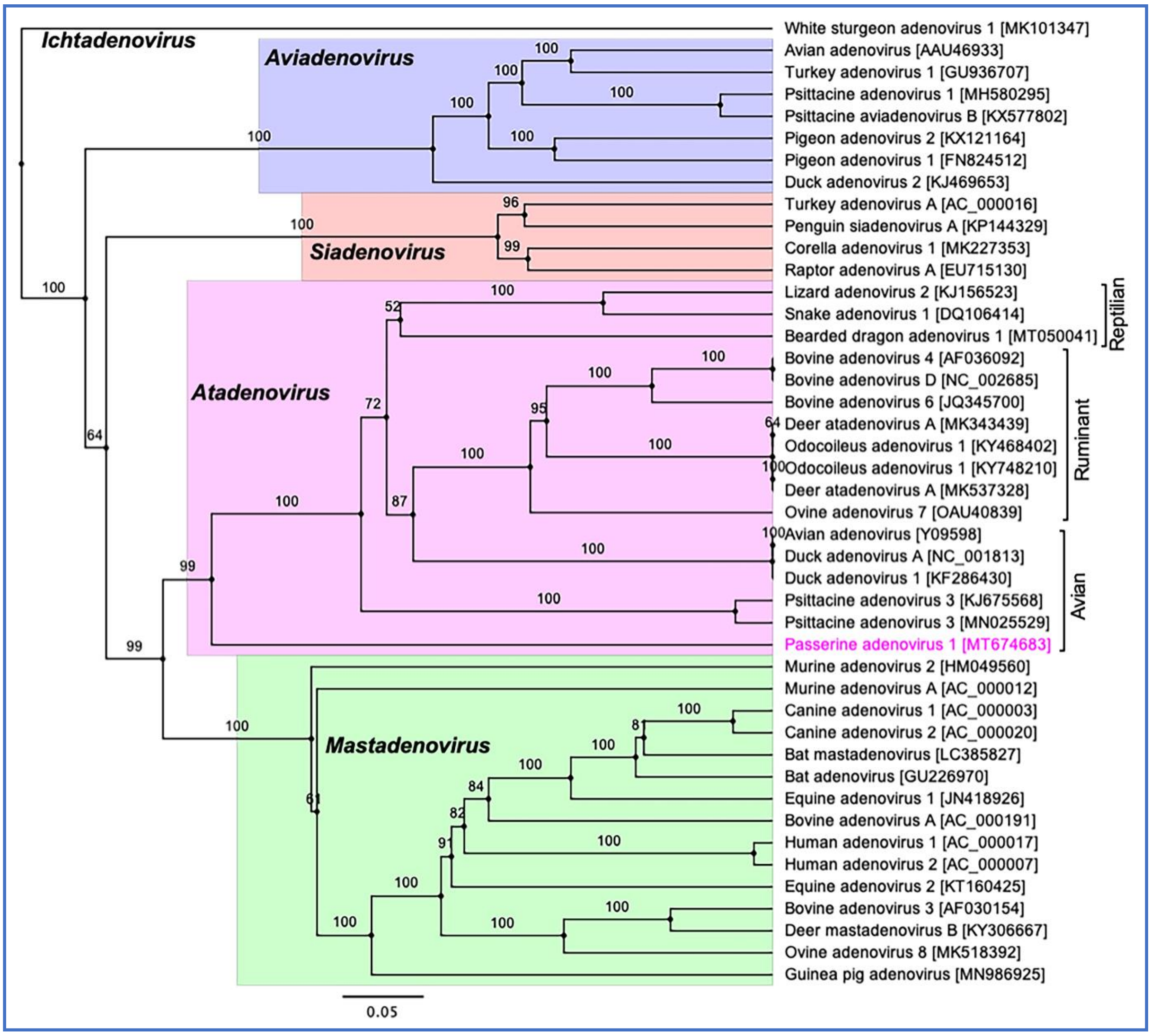

Figure S3. Phylogenetic tree showed the possible evolutionary relationship of novel passerine adenovirus 1 with other selected AdVs. Neighbor-Joining (NJ) tree was constructed using concatenated amino acid sequences of the complete DNA-dependent DNA polymerase, pTP, penton and hexon genes. Concatenated protein sequences were aligned with MAFTT (version 7.388) (Katoh and Standley, 2013) in Geneious (version 10.2.2) under the BLOSUM62 scoring matrix and gap open penalty =1.53. The gap >30 residues deleted from the alignments. The unrooted NJ tree was constructed using Jukes-Cantor genetic distance model with 1000 bootstrap resamplings in Geneious (version 10.2.2). The numbers on the left shown bootstrap values as percentages and the labels at branch tips refer to original AdVs species name followed by GenBank accession number in parentheses. The final tree was visualised with FigTree (version 1.4.4). The five official genera were highlighted as different background colours and novel passerine adenovirus 1 was shown as pink colour.

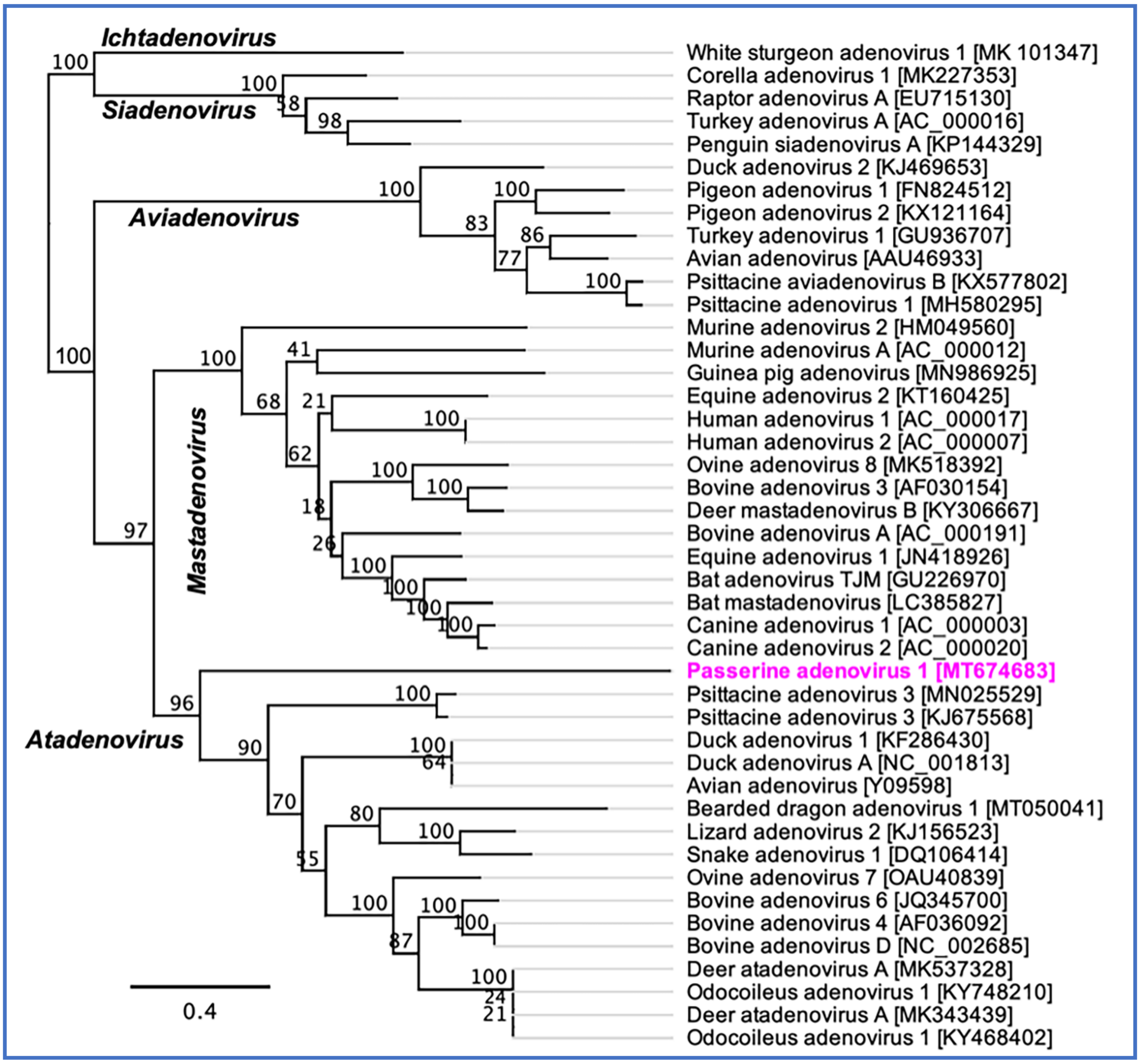

Figure S4. Phylogenetic tree showed the possible evolutionary relationship of novel passerine adenovirus 1 with other selected AdVs. Maximum likelihood (ML) tree was constructed using amino acid sequences of the complete DNA-dependent DNA polymerase gene. Selected protein sequences were aligned with MAFTT (version 7.450) (Katoh and Standley, 2013) in Geneious (version 10.2.2) under the BLOSUM62 scoring matrix and gap open penalty =1.53. The unrooted ML tree was constructed with PhyML (Guindon et al., 2010) under the LG substitution model, and 100 bootstrap resamplings were chosen to generate ML trees using tools available in Geneious (version 10.2.2). The numbers on the left shown bootstrap values as percentages and the labels at branch tips refer to original AdVs species name followed by GenBank accession number in parentheses. The novel passerine adenovirus 1 was shown as pink colour.

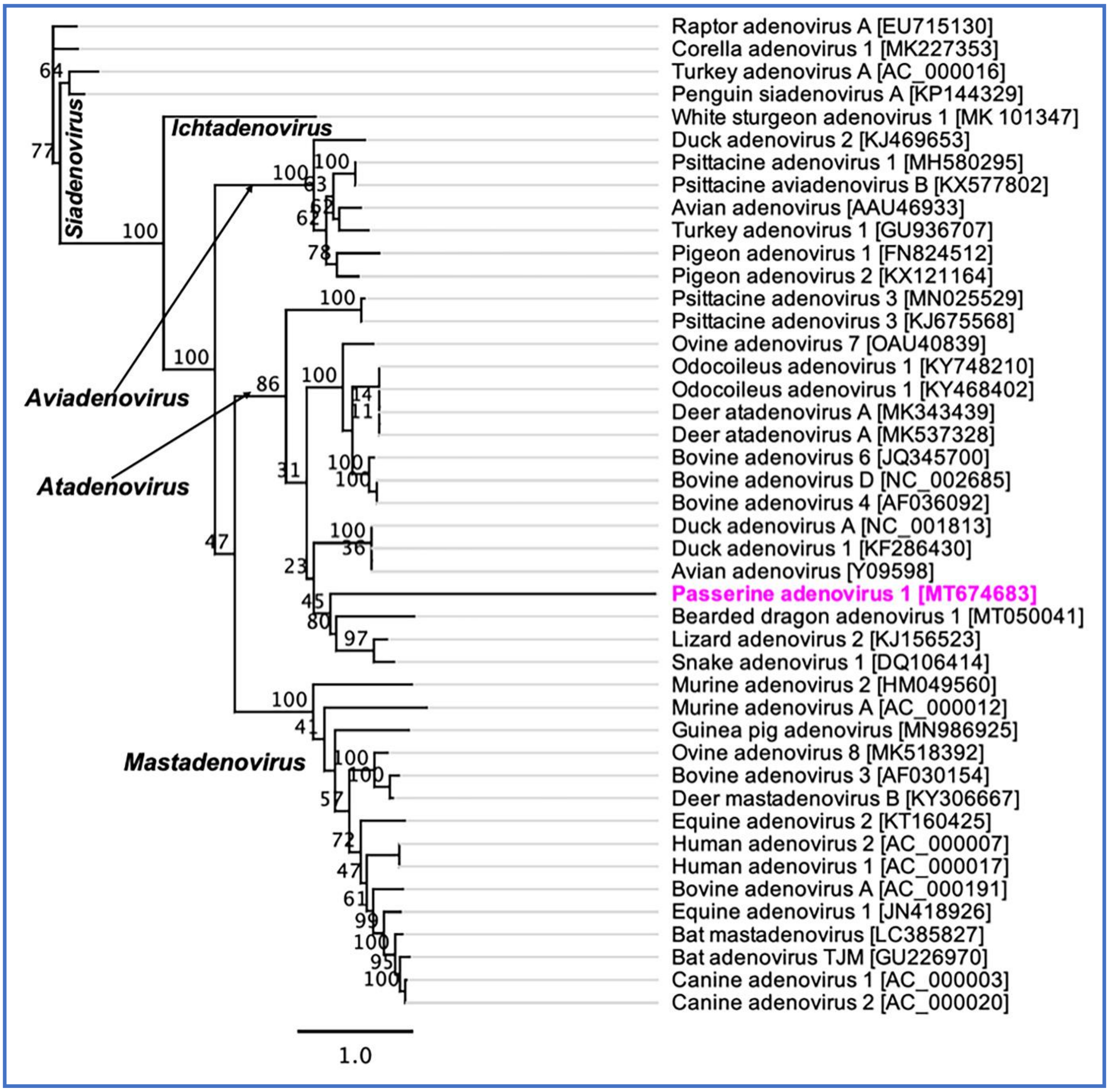

Figure S5. Phylogenetic tree showed the possible evolutionary relationship of novel passerine adenovirus 1 with other selected AdVs. Maximum likelihood (ML) tree was constructed using amino acid sequences of the complete pTP gene. Selected protein sequences were aligned with MAFTT (version 7.450) (Katoh and Standley, 2013) in Geneious (version 10.2.2) under the BLOSUM62 scoring matrix and gap open penalty =1.53. The unrooted ML tree was constructed with PhyML (Guindon et al., 2010) under the LG substitution model, and 100 bootstrap resamplings were chosen to generate ML trees using tools available in Geneious (version 10.2.2). The numbers on the left shown bootstrap values as percentages and the labels at branch tips refer to original AdVs species name followed by GenBank accession number in parentheses. The novel passerine adenovirus 1 was shown as pink colour.

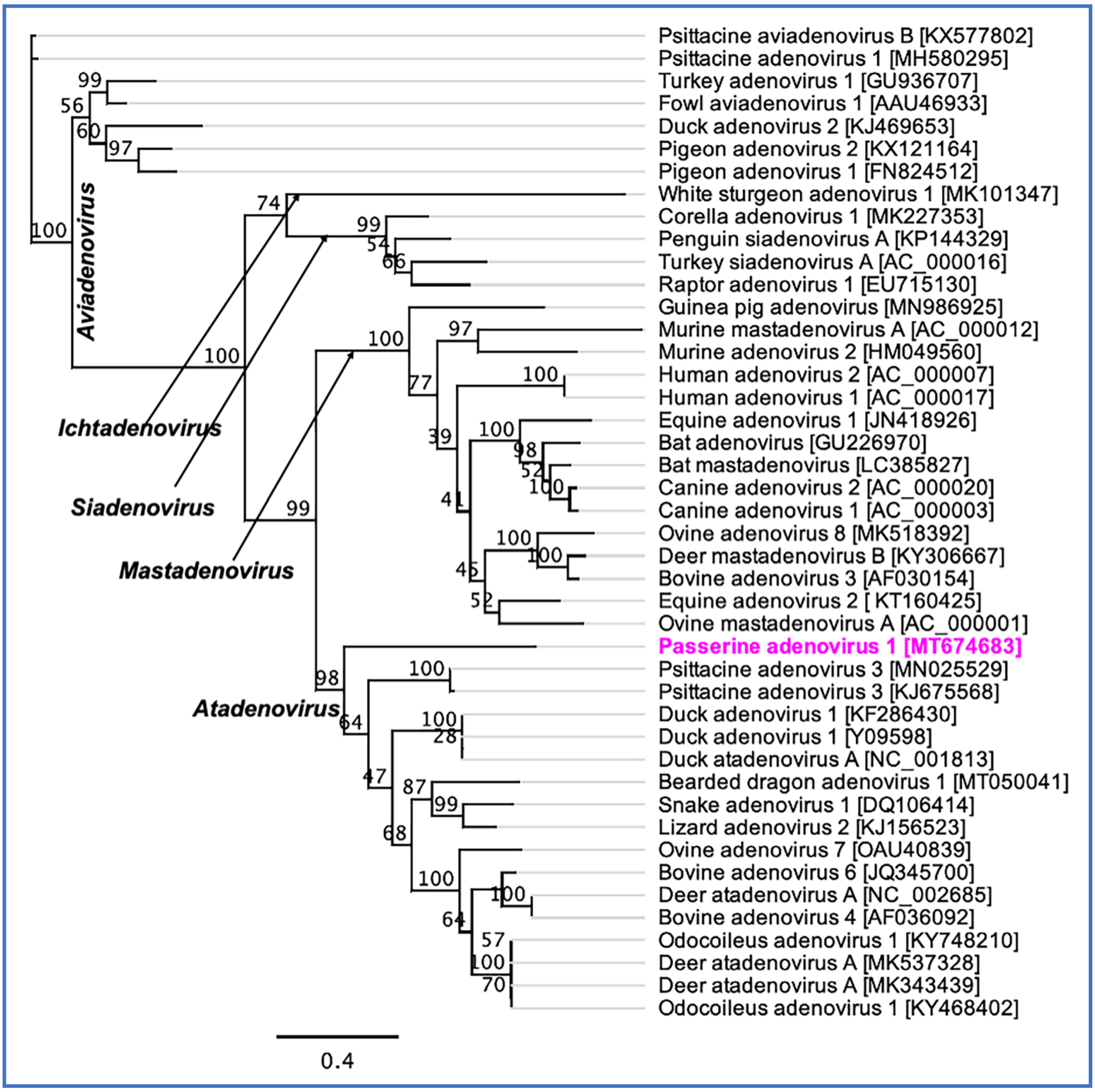

Figure S6. Phylogenetic tree showed the possible evolutionary relationship of novel passerine adenovirus 1 with other selected AdVs. Maximum likelihood (ML) tree was constructed using amino acid sequences of the complete penton gene. Selected protein sequences were aligned with MAFTT (version 7.450) (Katoh and Standley, 2013) in Geneious (version 10.2.2) under the BLOSUM62 scoring matrix and gap open penalty =1.53. The unrooted ML tree was constructed with PhyML (Guindon et al., 2010) under the LG substitution model, and 100 bootstrap resamplings were chosen to generate ML trees using tools available in Geneious (version 10.2.2). The numbers on the left shown bootstrap values as percentages and the labels at branch tips refer to original AdVs species name followed by GenBank accession number in parentheses. The novel passerine adenovirus 1 was shown as pink colour.

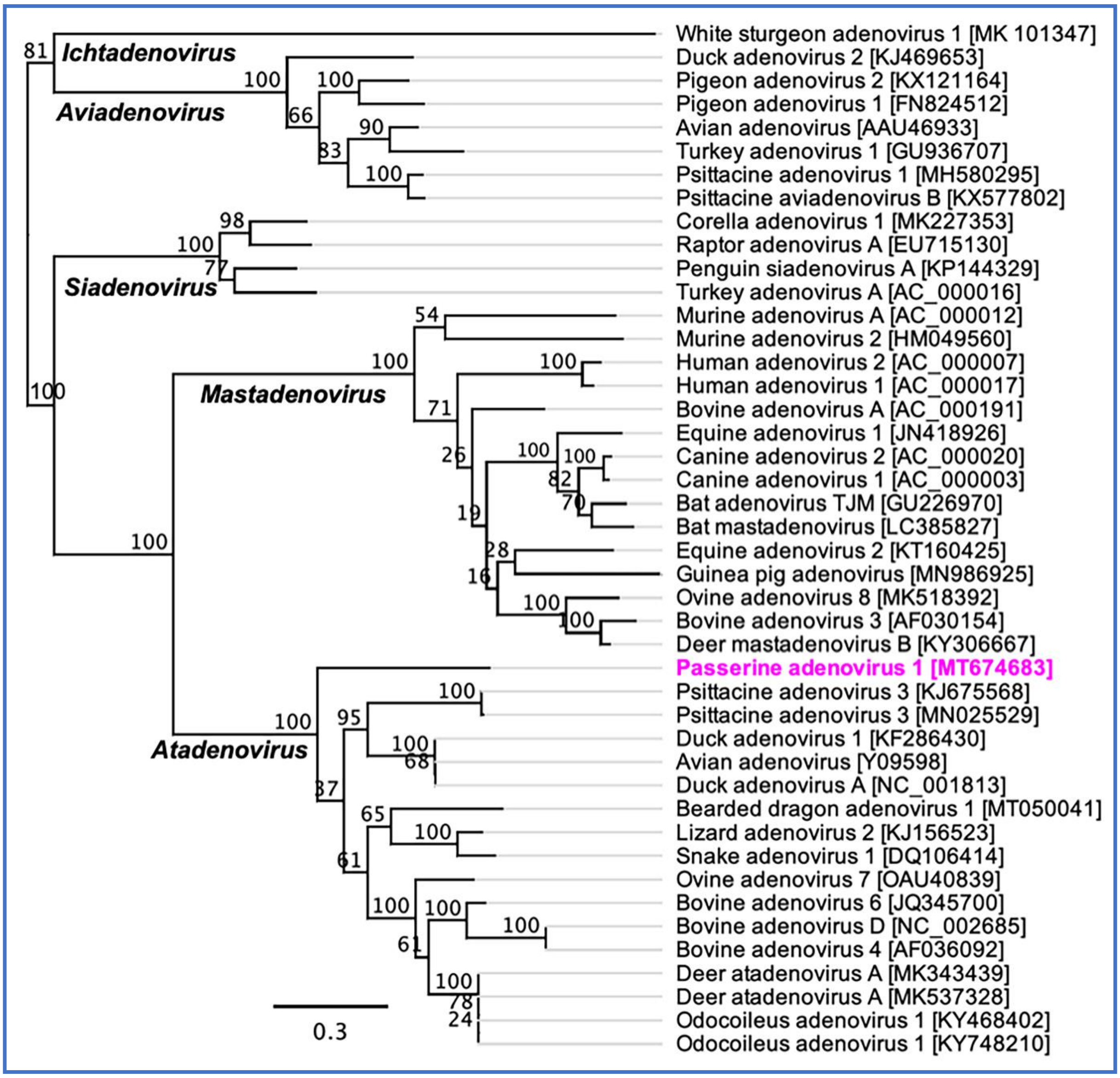

Figure S7. Phylogenetic tree showed the possible evolutionary relationship of novel passerine adenovirus 1 with other selected AdVs. Maximum likelihood (ML) tree was constructed using amino acid sequences of the complete hexon gene. Selected protein sequences were aligned with MAFTT (version 7.450) (Katoh and Standley, 2013) in Geneious (version 10.2.2) under the BLOSUM62 scoring matrix and gap open penalty =1.53. The unrooted ML tree was constructed with PhyML (Guindon et al., 2010) under the LG substitution model, and 100 bootstrap resamplings were chosen to generate ML trees using tools available in Geneious (version 10.2.2). The numbers on the left shown bootstrap values as percentages and the labels at branch tips refer to original AdVs species name followed by GenBank accession number in parentheses. The novel passerine adenovirus 1 was shown as pink colour.
